# Elucidating the Mechanism of Interactions Between Aminoglycosides and AuNPs: Why the Classical Colorimetric Assay May Falsely Report Aptamer Affinity

**DOI:** 10.3390/bios16070388

**Published:** 2026-07-17

**Authors:** Yaning Liang, Shiyi Fang, Zhuoer Chen, Yuzhuo Chen, Qingqing Yang, Xuelan Shu, Tao Le

**Affiliations:** Chongqing Key Laboratory of Conservation and Utilization of Freshwater Fishes, College of Life Sciences, Chongqing Normal University, Chongqing 401331, China; 2024110513044@stu.cqnu.edu.cn (Y.L.); 2025210513058@stu.cqnu.edu.cn (S.F.); 2025010513002@stu.cqnu.edu.cn (Z.C.); 2024110513031@stu.cqnu.edu.cn (Y.C.); 2024210513076@stu.cqnu.edu.cn (Q.Y.); 2025110513059@stu.cqnu.edu.cn (X.S.)

**Keywords:** AuNPs, aminoglycoside antibiotic, adsorption, aptamer, colorimetric assay

## Abstract

Gold nanoparticles (AuNPs) are widely used in aptasensors because of their high extinction coefficient and aggregation-dependent color differences. However, recent studies have indicated that nonspecific interactions between target molecules and AuNPs may dominate the detection signal rather than aptamer–target specific binding. This study systematically investigated the interactions between 13 aminoglycoside antibiotics and AuNPs. We found that all aminoglycoside antibiotics interacted strongly with AuNPs, considerably reducing their salt stability. Furthermore, methoxy polyethylene glycol thiol reversed AuNP aggregation induced by aminoglycoside antibiotics, indicating that it occurs at the secondary minimum. Using density functional theory, we analyzed the molecular structures and charge distribution characteristics of the aminoglycoside antibiotics, elucidating that they replace citrate ions on AuNP surfaces via a ligand exchange mechanism, thereby inducing aggregation. Additionally, both aptamer targets and complementary DNA struggled to desorb the aptamer (KAN6-1) from the AuNP surfaces. Our study demonstrates that the label-free colorimetric assay based on aggregation of unmodified citrate–AuNPs is neither suitable for characterizing the binding affinity of aminoglycoside aptamers nor viable for constructing corresponding colorimetric sensors to detect this class of antibiotics. Thus, researchers should incorporate mechanistic verification and rigorous controls when employing this system to ensure reliable results.

## 1. Introduction

Gold nanoparticles (AuNPs) are nanomaterials characterized by an extremely high extinction coefficient. This property enables direct visual observation at concentrations as low as nanomolar to picomolar levels, resulting in ultrasensitive visual detection [1,2]. Because of the surface plasmon resonance phenomenon, AuNPs appear red when dispersed and blue when aggregated. Because of this color change, they are widely used in the development of various biosensors [3,4,5,6]. The ability of AuNPs to adsorb oligonucleotides, thereby preventing salt-induced aggregation, has been utilized to develop label-free colorimetric sensors for DNA detection [7,8]. Subsequently, this method has been extended to aptamer–target detection, with the color change attributed to the specific binding of aptamers to targets. Currently, AuNP-based colorimetric aptasensor strategies are widely applied in fields such as pesticide residue analysis [9,10], food safety [11,12,13], environmental monitoring [14,15], and medical diagnosis [16,17].

Whelan et al. found in a 2017 study that for three representative proteins, varying degrees of pH-dependent aggregation occurred even in the absence of aptamers [18]. Recently, Liu et al. suggested that it is important to consider the interactions between target molecules and AuNPs. These interactions can alter the stability of AuNPs and affect how aptamers adsorb on their surfaces. Since 2019, their series of studies has revealed that target–AuNP interactions often dominate in this system [19,20,21,22,23,24,25,26]. Similarly, in 2022, Wang et al. noted that although citrate–AuNPs are extensively employed for marine toxin detection, potential nonspecific interactions with targets are frequently neglected. To address this, they developed tyrosine–AuNPs to construct more stable colorimetric sensors [27]. The following year, Liu et al. [28] and Yang et al. [29] separately examined the adsorptive behavior of tetracycline and four pesticides (acetamiprid, carbendazim, pymetrozine, and thiabendazole) toward AuNPs. Their further evaluation in label-free colorimetric detection methods demonstrated that all tested DNA sequences induced similar AuNP aggregation. These findings indicated that the observed color change originated not from specific aptamer–target interactions, but rather from nonspecific adsorption of the target molecules onto the AuNP surfaces. Our recent research similarly identified potent interactions between numerous antibiotics and AuNPs [30]. However, given that previous studies only qualitatively indicated the existence of the interactions, this study aimed to establish an optical quantitative index based on the degree of aggregation, in order to compare the ability of different aminoglycoside antibiotics (AAs) to destabilize AuNP stability in colloidal systems. Based on this, this study quantifies the strength of these interactions by determining the half-maximal aggregation concentration (AC_50_) of AAs that induce the aggregation of AuNPs. Collectively, these studies consistently indicate that in conventional label-free AuNP colorimetric assays, the target–AuNP interaction is the most important factor in the signal, suggesting that AuNPs are unsuitable as carriers for certain aptasensors. Although this phenomenon has gradually been revealed, the underlying molecular mechanism of the target–AuNP interaction remains to be investigated. Elucidating the operating mechanism is crucial for predicting the types of target molecules prone to interacting with AuNPs, thereby preventing the misuse of such systems in aptamer selection, affinity characterization, and biosensor development.

This study reveals strong nonspecific interactions between 13 AAs and AuNPs. AAs displace citrate ions on AuNPs via ligand exchange, weakening electrostatic stability, and causing reversible nanoparticle aggregation with a pronounced red shift in the plasmon coupling absorption peak. Density functional theory (DFT) calculations suggest that the lone pair electrons from the amino nitrogen atoms in AAs can form strong coordination interactions with the Au surfaces. Moreover, once adsorbed on AuNP surfaces, the aptamer is difficult to displace by its targets or complementary DNA (cDNA), and its modification considerably inhibits the response of AuNPs to AAs, which is detrimental to the generation of detection signals. Consequently, we propose that the label-free colorimetric assay based on the aggregation of unmodified citrate-AuNPs may be unsuitable for characterizing the binding affinity of aminoglycoside aptamers, nor for developing corresponding colorimetric sensors. This is primarily due to two factors: first, excessively strong adsorption between aptamers and AuNPs hinders the desorption process; and second, nonspecific interactions between the targets and AuNPs readily interfere with detection results. This could lead to the reporting or application of aptamers with low or even ineffective affinity, impeding their subsequent development and utilization.

## 2. Materials and Methods

### 2.1. Chemicals

Trisodium citrate, chloroauric acid (HAuCl_4_), methoxy polyethylene glycol thiol (mPEG–SH), bis (p-sulfonatophenyl) phenyl-phosphine dihydrate dipotassium salt (BSPP), sodium chloride (NaCl), thioflavin T (ThT), 2-morpholinoethanesulfonic acid (MES), potassium ferricyanide (K_3_[Fe(CN)_6_]), and all other chemical reagents were purchased from Sangon Biotech (Shanghai, China). Tobramycin (TOB), kanamycin (KAN), neomycin (NEO), amikacin (AMI), sisomicin (SIS), ribostamycin (RIB), gentamicin (GEN), streptomycin (STR), dihydrostreptomycin (DIH), netilmicin (NET), paromomycin (PAR), apramycin (APR), and spectinomycin (SPE) were purchased from Aladdin (Shanghai, China). The aptamer KAN6-1 used in this study was obtained from Liu’s team [31], and its specific sequence is provided in Table S1. Ultrapure water (18.2 MΩ·cm resistivity at 25 °C) was used throughout this study.

### 2.2. Preparation of Citrate–AuNPs and BSPP–AuNPs

The citrate–AuNPs were prepared as follows. First, 1 mL of 1% HAuCl_4_ (1 g HAuCl_4_ dissolved in 100 mL of ddH_2_O) was added to 99 mL of ddH_2_O. After mixing uniformly, the solution was stirred at 300 °C and 250 rpm on a magnetic stirrer (BIOBASE, Jinan, China). Subsequently, 2.25 mL of freshly prepared 1% trisodium citrate (1 g trisodium citrate dissolved in 100 mL of ddH_2_O) was rapidly added, and heating continued until the solution turned wine-red. The solution was cooled to room temperature, then concentrated 4-fold and stored at 4 °C for later use.

BSPP–AuNPs were synthesized from citrate–AuNPs. A magnetic stirrer was used to stir 100 mL of the citrate–AuNP solution at 300 rpm while 1 mL of a 30 mg/mL BSPP solution was added dropwise. After complete addition, the mixture was stirred for 15 min and then incubated overnight at 4 °C. The mixture was then centrifuged at 15,000 rpm for 15 min, and the supernatant was discarded. To ensure complete citrate removal, the precipitate was resuspended in an equal volume of a 100 µM BSPP solution and repeated three time. Finally, the BSPP–AuNPs were concentrated 4-fold and stored at 4 °C for future use.

### 2.3. Transmission Electron Microscopy

Citrate–AuNPs were dispersed in ddH_2_O via sonication. Subsequently, samples were dispersed over copper grids coated with an ultrathin carbon film. After drying, observations were conducted using a JEM-F200 transmission electron microscope (JEOL, Tokyo, Japan) operating at 200 kV. The AuNPs synthesized in this study had a diameter of ~15 nm (Figure S1).

### 2.4. Interaction Between AuNPs and AAs

In a 96-well plate, 80 µL of ddH_2_O was mixed with 100 µL of 4-fold concentrated AuNPs. Subsequently, 20 µL of 10 mM AAs (TOB, KAN, NEO, AMT, SIS, RIB, GEN, STR, DIH, NET, PAR, APR, and SPE) was added to ensure complete aggregation of the AuNPs. After thorough mixing, the ultraviolet–visible (UV–Vis) spectrum was recorded from 400 to 800 nm. The results showed that treatment with AAs, the absorption peak of the AuNPs exhibited a red shift, indicating the occurrence of plasmon coupling between the AuNPs.

For five of the AAs (AMI, NET, NEO, SIS, and TOB), 450 µL of ddH_2_O was mixed with 500 µL of 4-fold concentrated AuNPs in a 48-well plate. To achieve a final concentration of 1 µM, 50 µL of 20 µM AAs was then titrated stepwise into the mixture. Changes in absorbance during the titration were monitored using a microplate reader. For the remaining seven AAs (APR, GEN, PAR, RIB, STR, DIH, and KAN), 50 µL of 200 µM AAs was titrated stepwise into a 950 µL mixture until a final concentration of 10 µM was achieved. The absorbance changes were recorded simultaneously. For SPE, titration was performed using a 20 mM solution until a final concentration of 1 mM was attained. All experiments were carried out in triplicate. Nonlinear regression analysis was performed using the four-parameter logistic (4PL) model with OriginPro 2024 (10.1) software, from which the AC_50_ were calculated. Data were presented as mean ± SD (*n* = 3). The lower the AC_50_ value, the greater the potency for inducing AuNP aggregation.

To investigate the effect of AAs on the salt stability of AuNPs, 80 µL of ddH_2_O, 100 µL of 4-fold concentrated AuNPs, and 20 µL of AAs were added to a 96-well plate. The following concentrations of AAs were used to ensure that AuNPs did not aggregate: 300 nM KAN, 150 nM AMI, 140 nM APR, 500 nM DIH, 100 nM GEN, 100 nM NEO, 90 nM NET, 100 nM PAR, 200 nM RIB, 85 nM SIS, 40 µM SPE, 400 nM STR, or 100 nM TOB. UV–Vis spectra were recorded from 400 to 800 nm. Next, NaCl was introduced into the mixture to a final concentration of 30 mM. After thorough mixing, the spectrum of each group was measured again. Finally, to investigate the aggregation behavior further, additional NaCl was added to increase the final concentration to 60 mM, inducing complete aggregation of the AuNPs in the control group. Then the spectra of all groups were measured again.

### 2.5. Interactions Between AuNPs and Deprotonated AAs

To inhibit the protonation of AAs, the pH of the AuNP solution was adjusted to 12. Then, according to the method described in Section 2.4, 50 µL of 20 mM AAs was titrated stepwise into 950 µL of AuNPs (PH 12). The absorbance changes during titration were monitored using a microplate reader. The experimental data were fitted using OriginPro 2024 (10.1) to calculate the AC_50_, allowing us to assess the interaction strength between AuNPs and deprotonated AAs at pH 12.

### 2.6. mPEG–SH Reverses AuNP Aggregation Induced by AAs

This study evaluated the reversibility of AuNP aggregation induced by AAs via introducing mPEG–SH. Initially, 180 µL of AuNPs was mixed with 20 µL of 2.5 µM KAN in a 96-well plate. Then, 1 mM mPEG–SH of 550 Da, 3000 Da, or 10,000 Da was added, resulting in final mPEG–SH concentrations of 2.5, 5, 10, 15, and 20 µM in the system. The color changes in the system were recorded using a digital camera, revealing that 3000 Da mPEG–SH exhibited the optimal reversal effect on AuNP aggregation induced by AAs. Therefore, 3000 Da mPEG–SH was selected for all subsequent experiments.

To assess the ability of mPEG–SH to reverse AuNP aggregation induced by AAs, the following three experimental groups were set up. Group 1: 180 µL of 4-fold concentrated AuNPs and 20 µL of 500 µM AAs were added to a 96-well plate to induce complete AuNP aggregation, with the control group using an equal volume of ddH_2_O instead of the AAs. Group 2: Based on Group 1, 20 µL of 300 mM NaCl was added. Group 3: Based on Group 1, 20 µL of 600 mM NaCl was added. Each well was thoroughly mixed using a pipette before adding 10 µL of 1 mM 3000 Da mPEG–SH, followed by additional mixing. Images of each group were captured before and after adding mPEG–SH.

### 2.7. Computer Experimental Section

The structures of all AAs were drawn using ChemDraw (19.0.0.22), and the pKa values of their respective functional groups were predicted employing the MolGpKa online service [32].

The three-dimensional (3D) structures of the AAs were drawn using Chem3D (19.0.0.22). Subsequently, DFT calculations were performed using the ORCA 6.0.1 software, with all calculations conducted under the SMD water model [33]. This included geometry optimization, frequency calculations, and single-point energy calculations. The wB97X functional [34] with D3 dispersion correction and the def2-SVP basis set were used for geometry optimization and frequency calculations, ensuring no imaginary frequencies were present, followed by single-point energy calculations on the optimized 3D structures. The resulting wavefunction files were processed using Multiwfn 3.8 (dev) to calculate the molecular electrostatic potential (ESP), π-electron distribution, and highest occupied molecular orbital (HOMO)–lowest unoccupied molecular orbital (LUMO) for the AAs [35].

### 2.8. Aptamer Affinity Assay

The aptamer powder was centrifuged at 4000 rpm for 30 s to prevent sample loss after opening the lid. An appropriate volume of MES buffer (10 mM MES, 50 mM NaCl, and 2 mM MgCl_2_, pH = 6) was then added, followed by vortex mixing and centrifugation to ensure complete dissolution. The solution was first incubated in a 95 °C metal bath for 10 min, then rapidly transferred to an ice bath for 10 min, and finally equilibrated at room temperature for 10 min.

ThT was employed as a fluorescent probe to determine the affinity of KAN6-1. A mixture of 313 µL of MES buffer, 7 µL of 10 µM aptamer, and 10 µL of 200 µM ThT was prepared in a 96-well plate. Then, 20 µL of 87.5 µM corresponding target was gradually titrated. The changes in fluorescence intensity during titration were recorded using a microplate reader. All experiments were carried out in triplicate. Nonlinear regression analysis was performed using the four-parameter logistic (4PL) model with OriginPro 2024 (10.1) software, from which the K_d_ were calculated. Data were presented as mean ± SD (*n* = 3).

### 2.9. Desorption Kinetics Experiment of AuNP-Adsorbed Aptamer

Time-scan fluorescence measurements of the interaction process between aptamer-functionalized AuNPs, the targets, and cDNA were performed using a fluorescence spectrophotometer (Drawell, Chongqing, China). Into a 4 mL standard cuvette, 950 µL of 4-fold concentrated AuNPs and 10 µL of 10 µM FAM-labeled aptamer were added. After premixing, time-scan recording was initiated at an excitation wavelength of 497 nm and an emission wavelength of 518 nm. At 5 min during the scan, 40 µL of 100 nM target or 40 µL of 2.5 µM cDNA was added to the system, and the change in fluorescence intensity was continuously recorded. At 10 min during the scan, 50 µL of K_3_[Fe(CN)_6_] was added to etch the AuNPs, releasing the FAM-labeled aptamer adsorbed on the AuNPs, and scanning was continued for an additional 20 min.

## 3. Results and Discussion

### 3.1. AAs Directly Induce AuNP Aggregation

**Figure 1:**
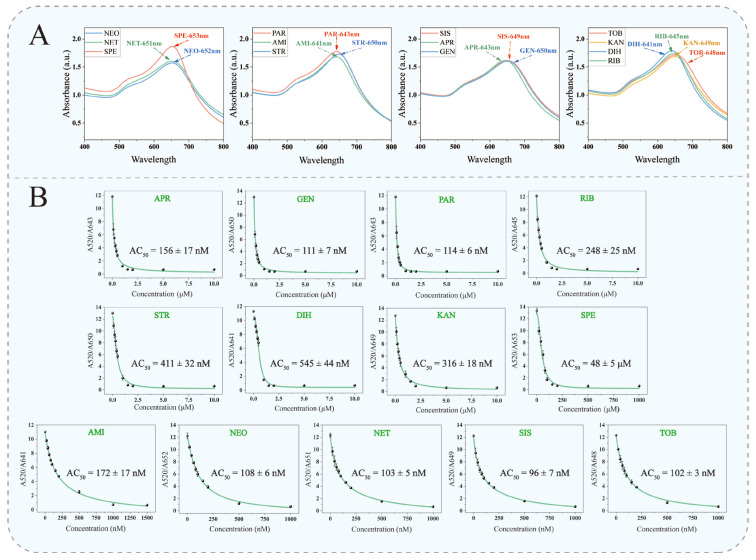
Direct induction of AuNP aggregation by AAs. (**A**) UV–Vis spectra of AuNPs in the 400–800 nm wavelength range after complete aggregation was induced by the adsorption of 1 mM AAs. The molecular dimensions of the AAs cause plasmon coupling effects, leading to a red shift in the absorption peak. (**B**) The half-maximal aggregation concentration (AC_50_) of 13 AAs for AuNPs was determined via titration.

Previous studies have shown that various small molecules can interact strongly with AuNPs, thereby affecting their stability. Therefore, we systematically investigated the interactions of 13 AAs with AuNPs. When the concentration of AAs reached 1 mM, AuNP aggregation was directly induced, causing the solution color to change from red to blue. The UV–Vis absorption spectra recorded under these conditions showed a substantial red shift in the absorption peak within the 400–800 nm range (Figure 1A). Furthermore, the extent of the absorption peak red shift differed among AAs, which may be related to differences in molecule size. These results indicate that AAs adsorbed on AuNP surfaces, inducing a plasmon coupling effect, which ultimately led to AuNP aggregation.

To quantitatively compare the interaction strengths of the different AAs, we measured their AC_50_ with AuNPs (Figure 1B). The results revealed that, among the 13 AAs, SPE exhibited the weakest interaction with AuNPs (AC_50_ of 49 ± 5 μM), whereas TOB and SIS had strong interactions with AuNPs (AC_50_ values of 102 ± 3 nM and 96 ± 7 nM, respectively).

**Figure 2:**
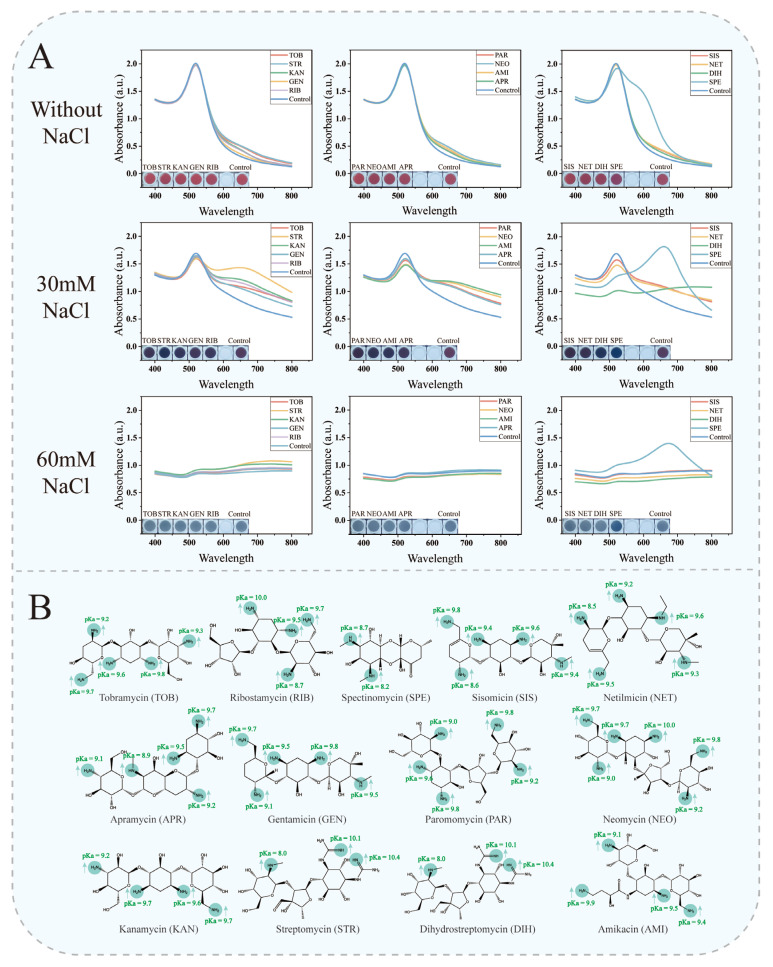
(**A**) Photographs and UV–Vis spectra of AuNPs after interaction with AAs in the presence of 0, 30, or 60 mM NaCl. (**B**) Predicted pKa of the amino groups in AAs. Protonation is predicted to occur at pH = 7.

Additionally, the adsorption of AAs affected the salt stability of AuNPs. Under 30 mM NaCl conditions, the control AuNPs showed minimal change; however, AuNPs treated with specific concentrations of AAs began to aggregate (Figure 2A). When the NaCl concentration was increased to 60 mM, the AuNPs treated with a higher SPE concentration remained light blue. In contrast, the control group and the AuNPs treated with lower concentrations of AAs aggregated completely, resulting in a light gray appearance. These results indicate that higher SPE concentrations inhibited salt-induced AuNP aggregation. The corresponding UV–Vis spectra further confirmed this phenomenon. After treatment with high-concentration SPE, an absorption peak near 650 nm remained visible; however, it was absent in the control and other groups, indicating that the high concentrations of AAs adsorbed on the AuNP surfaces provided a protective effect, preventing their complete aggregation under high-salt conditions.

The above results indicate that all 13 AAs could directly induce AuNP aggregation. Furthermore, the adsorption of AAs onto the AuNP surfaces affected the salt stability of the AuNPs.

### 3.2. Protonation Promotes the Adsorption of Certain AAs on AuNPs

**Figure 3:**
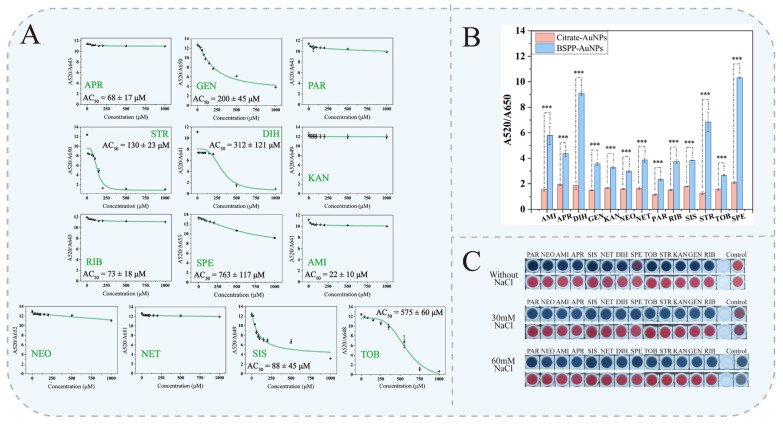
(**A**) The half-maximal aggregation concentration (AC_50_) was determined by titrating 50 µL of 20 mM AAs into 950 µL of AuNPs at pH 12. (**B**) Resistance of citrate-AuNPs and BSPP-AuNPs to 13 AAs. Antibiotic concentrations just sufficient to induce aggregation of citrate-AuNPs were used. Data are shown as mean ± SD. Statistical analyses were performed using OriginPro 2024 (10.1) software via one-way ANOVA with Fisher’s test. In the figure, *** *p* < 0.001 indicate extremely significant differences between groups. (**C**) Under different NaCl concentrations, aggregation of AuNPs induced by AAs and subsequent redispersion after adding mPEG-SH (3000 Da). The figure is vertically arranged into three panels (top, middle, and bottom), corresponding to 50 µM AAs alone, 50 µM AAs + 30 mM NaCl, and 50 µM AAs + 60 mM NaCl, respectively. Within each panel, the upper and lower rows show the states before and after mPEG-SH addition, respectively.

AAs typically comprise multiple amino groups with relatively high pKa values, which are prone to protonation under neutral conditions, conferring a positive charge to the AAs (Figure 2B). In this form, AAs may function similarly to salt ions, inducing AuNP aggregation via charge screening. To investigate the effect of protonation on the interaction between AAs and AuNPs, we adjusted the solution pH to 12, which suppresses the protonation of AAs. At pH 12 and a concentration of 1 mM, substantial AuNP aggregation was not induced by APR, PAR, RIB, KAN, AMI, NEO, or NET (Figure 3A). SIS, GEN, and SPE caused only slight aggregation, with AC_50_ values increasing by 1798-fold, 919-fold, and 16-fold, respectively, compared to pH 7. In contrast, STR, DIH and TOB induced complete AuNP aggregation even at elevated concentrations, with their AC_50_ values increasing by 317-fold, 574-fold and 5654-fold, respectively, relative to pH 7. These results indicate that although protonation can promote the interaction of some AAs with AuNPs, it is not the dominant factor. Under neutral pH conditions, the positive charge carried by protonated AAs can electrostatically attract citrate–AuNPs (negatively charged), thereby facilitating the approach and adsorption of AAs on AuNP surfaces. However, when the pH is markedly increased to 12, several factors impede the interaction of AAs and AuNPs. First, greater deprotonation of citrate ions increases the negative surface charge density of AuNPs, improving colloidal stability considerably. This may be one reason why some AAs fail to induce AuNP aggregation under these conditions. Second, when the pH increases to 12, the amino groups on some AAs deprotonate and acquire a negative charge, resulting in electrostatic repulsion with the negatively charged AuNPs, making it difficult for them to approach and adsorb.

### 3.3. Ligand Exchange: The Cause of AuNP Aggregation Induced by AAs

The 13 AAs induced aggregation of AuNPs and affected their salt stability to varying degrees (Figure 2A). Under 30 mM NaCl conditions, the control AuNPs showed no substantial change, whereas AuNPs treated with AAs had already begun to aggregate. When the NaCl concentration was increased to 60 mM, the control group aggregated completely and appeared light gray. In contrast, AuNPs with 40 μM SPE exhibited a light blue color and retained an absorption peak near 650 nm. This suggests that AAs may partially displace the citrate ions that coat the AuNP surfaces, leading to a lower surface potential and a weaker charge balance, thereby lowering the critical concentration at which they resist salt-induced aggregation. Therefore, at the same NaCl concentration (30 mM), AuNPs treated with certain concentrations of AAs had already aggregated, whereas the control group remained dispersed. When the NaCl concentration was further increased to 60 mM, the AuNPs treated with a high SPE concentration exhibited a different color and UV–Vis spectral features than the control group. This may be because the higher SPE concentration displaced more citrate ions from the AuNP surfaces via ligand exchange and created a degree of steric hindrance on the particle surface, thereby partially inhibiting the physical aggregation induced by NaCl and exerting a protective effect on the AuNPs. Based on these results, we hypothesized that AAs induce AuNP aggregation via a ligand exchange mechanism, affecting salt stability.

To verify that AAs affect AuNP stability via ligand exchange, we synthesized BSPP–AuNPs (Figure S2A). The phosphorus atom in BSPP has a much stronger coordination ability with AuNPs than citrate; therefore, BSPP–AuNPs were expected to exhibit greater resistance to AAs. After adding AAs, the color of citrate–AuNPs changed from red to purple or even blue, whereas BSPP–AuNPs showed almost no change, revealing a substantial difference between the two systems (Figure 3B and Figure S2B). Moreover, at the same concentration, the various AAs had different effects on BSPP–AuNPs. SPE, DIH, and STR had the weakest interactions with BSPP–AuNPs, followed by AMI, whereas TOB, KAN, and NEO showed stronger interactions. The above results further support the existence of a ligand exchange mechanism.

**Figure 4:**
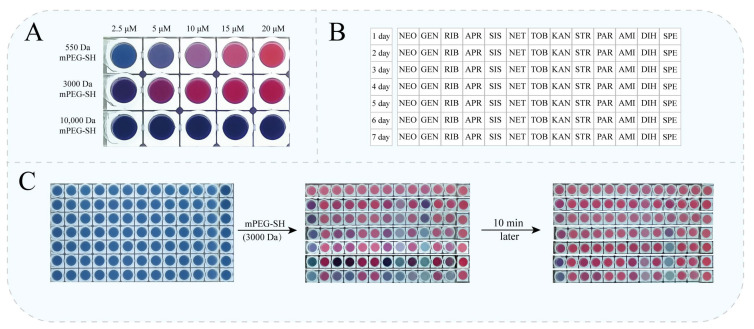
(**A**) The reversal effect of mPEG–SH at varying concentrations and molecular weights on AuNP aggregation induced by 2.5 µM KAN. (**B**) Corresponding table of AAs added to each well, matching all images in (**C**). (**C**) Images showing the immediate reversal effect and the reversal effect 10 min after adding mPEG–SH (3000 Da) to AuNPs containing 1 mM AAs that have been standing for 1–7 days (from top to bottom: stood for 1–7 days, respectively).

According to Derjaguin–Landau–Verwey–Overbeek (DLVO) theory, AuNP stability is governed by a balance between van der Waals attraction and electrostatic repulsion, corresponding to two distinct aggregation modes. If aggregation occurs at the primary minimum, particles are in direct contact, forming irreversible physical aggregation; if aggregation occurs at the secondary minimum, particles remain at a certain distance, forming weaker and reversible aggregation. To investigate which mode AuNP aggregation induced by AAs belongs to, we examined the reversibility of AuNP aggregation induced by AAs via introducing mPEG–SH. First, we employed mPEG–SH with different molecular weights (550, 3000, and 10,000 Da) to attempt to reverse AuNP aggregation induced by AAs, aiming to identify the molecular weight with the optimal reversal effect (Figure 4A). The results showed that under different concentration conditions, 3000 Da mPEG–SH had the best reversal effect on AuNP aggregation induced by AAs, followed by 550 Da, whereas 10,000 Da mPEG–SH exhibited almost no reversal effect. This phenomenon may be related to the gaps between AuNPs in the aggregated system: the small molecular size of AAs results in limited gaps between AuNPs after aggregation. Excessively high molecular weight mPEG–SH (e.g., 10,000 Da) struggles to enter these gaps and undergo effective ligand exchange on the AuNP surfaces, thus failing to achieve redispersion. Therefore, we selected 3000 Da mPEG–SH for subsequent studies. Subsequently, in a system where aggregation was directly induced by high concentrations of AAs, the addition of mPEG–SH (3000 Da) readily redispersed the AuNPs. The aggregation induced by AAs in the presence of 30 mM NaCl was also reversed by mPEG–SH (3000 Da). Under 60 mM NaCl conditions, AuNPs in all groups aggregated. Although mPEG–SH (3000 Da) did not reverse aggregation in the control group, it did reverse AuNP aggregation induced by AAs (Figure 3C). Additionally, we examined the time-dependent reversal effect of mPEG–SH on AuNP aggregation induced by AAs (Figure 4B,C). After adding AAs to AuNPs to induce aggregation, the system was allowed to stand for 1–7 days before being treated with 3000 Da mPEG–SH. The results showed that as the standing time increased, the reversal effect of mPEG–SH gradually diminished. This may be because the molecular size of AAs is much smaller than that of AuNPs. Over time, some AuNPs gradually transitioned from reversible secondary minimum aggregation to irreversible primary minimum aggregation, leading to a substantial decrease in the reversal effect of mPEG–SH. These results indicate that AuNP aggregation induced by AAs occurs at the secondary minimum, whereas NaCl-induced aggregation occurs at the primary minimum. This suggests that the steric hindrance generated by AAs adsorbed on the AuNP surfaces prevents direct particle contact, maintaining a reversible aggregated state, which is further supported by the plasmon coupling phenomenon resulting from AuNP aggregation induced by AAs. However, over an extended time, the state of some AuNPs gradually shifts toward irreversible physical aggregation, leading to a weakened reversal effect of mPEG–SH.

At this point, we can conclude that AAs adsorb onto the AuNP surfaces via ligand exchange and induce aggregation. This aggregation occurs at the secondary minimum. Owing to their molecular size, AAs cause steric hindrance between particles, preventing direct contact. However, because AAs are much smaller than the radius of AuNPs, plasmon coupling occurs, resulting in a solution color change and spectral red shift.

### 3.4. Hypothesis on the Interaction Mechanism Between AAs and AuNPs

To better understand the underlying interaction mechanism between AAs and AuNPs, we employed DFT to analyze the molecular structural characteristics and electron distribution of the 13 AAs. We examined the ESP, π-electron distributions, and frontier molecular orbitals to predict their potential adsorption sites and modes.

AAs comprise different numbers of amino groups, which may influence the strength of their interaction with AuNPs. Among them, SPE, with only two amino groups, exhibited the weakest interaction, with a AC_50_ of 48 ± 5 μM. STR and DIH, each with three amino groups, had AC_50_ values of 411 ± 32 nM and 545 ± 7 nM, respectively. Conversely, other AAs with four or five amino groups showed even lower AC_50_ values, indicating stronger interactions with AuNPs (Figure 1B).

**Figure 5:**
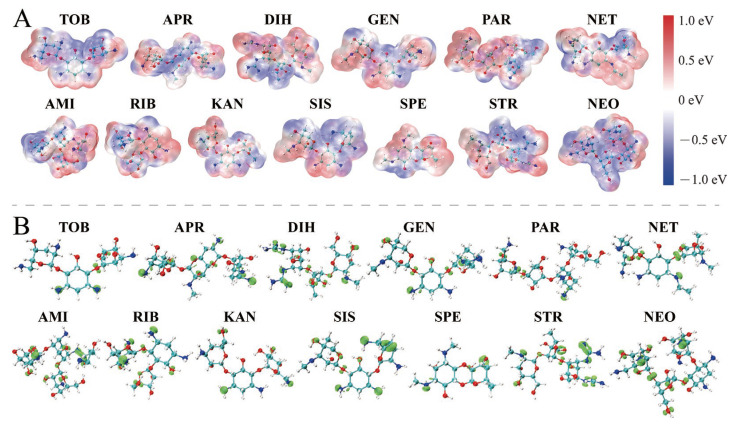
(**A**) Surface electrostatic potential distribution of 13 AAs calculated using DFT. Negative potential (blue) indicates regions of high electron density. (**B**) π-electron distribution of the 13 AAs.

Our DFT calculation results further demonstrate that, for AAs, the amino groups bearing multiple lone pair electrons are the primary functional groups responsible for their adsorption onto AuNPs. The ESP revealed substantial regions of negative electrostatic potential near some amino groups of the AAs (Figure 5A). This indicates the presence of abundant lone pair electrons on these amino groups, which demonstrates a strong electron-donating capability. Au, being a typical electrophilic atom, has a strong tendency to accept electrons. Therefore, the lone pair electrons from the amino groups can form strong σ-coordination bonds with Au. Analysis of the π-electron distribution (Figure 5B) showed a substantial electron cloud concentration around the amino nitrogen atoms, further confirming the high reactivity of these sites. Furthermore, based on frontier orbital theory, we analyzed the outermost electron distribution of AAs. The HOMO is primarily occupied by the amino sugar moiety and some amino groups (Figure S3). Although certain carbon and oxygen atoms on the amino sugar ring also contribute to the HOMO, the primary contributors are the amino groups.

In our previous study, we proposed that the presence of amino groups is not the sole determinant of AuNP adsorption and aggregation; rather, the key factor is the conjugation state of the lone pair electrons on the amino groups. Through ESP, electron localization function (ELF), and π-electron distribution analyses, we found that when the lone pair electrons of amino groups are conjugatively delocalized with adjacent carbonyls or aromatic rings (as in urea, alanine, and pyrimidine), their coordination ability is significantly weakened, providing only weak Au–π interactions. Conversely, if the lone pair electrons remain unconjugated (as in AAs, imidazole, and pyridine), they can form strong σ-coordination bonds with Au, thereby directly inducing AuNP aggregation at low concentrations. This is highly consistent with our current computational results. In addition, in our previous study, we also examined the effects of glucose and sucrose on the stability of AuNPs, in order to clarify the specific contributions of other groups in the molecules besides amino groups. Both control experiments and DFT calculations demonstrated that in glucose and sucrose, the regions of negative electrostatic potential are mainly concentrated around the oxygen atoms, and the dominant contribution to their HOMO energy levels also comes from the oxygen atoms. Meanwhile, Fukui function f^−^ analysis revealed that in AAs, the amino groups and some oxygen atoms are the main contributors, whereas the contributions from carbon atoms in the amino sugar ring are significantly reduced. For glucose and sucrose, oxygen atoms are likewise the principal contributors to the Fukui function f^−^ [30]. These results effectively exclude the contribution of other groups and confirm that amino groups bearing multiple lone pair electrons are the primary functional moieties responsible for the adsorption of AAs onto AuNPs, which further supporting our conclusions.

Based on the above results, we can conclude that for AAs, amino groups with multiple lone pair electrons are the main functional groups for adsorption onto AuNPs. This strong electron-donating ability ensures the formation of stable σ-coordination bonds between AAs and the Au surface, thereby providing a theoretical basis for the experimentally observed capacity to induce AuNP aggregation.

### 3.5. AAs and cDNA Cannot Desorb the Aptamer from the AuNP Surfaces

In previous studies, AuNP colorimetric aptasensor design has typically employed two strategies. The first involves preincubating the aptamer with the target to form a complex, which is then added to the AuNPs. Ideally, the conformational change induced by target binding make it difficult for aptamers to adsorb onto the AuNP surfaces. The subsequent addition of NaCl via charge screening induces rapid aggregation of the unprotected AuNPs, resulting in a color change from red to purple or blue. The second strategy involves pre adsorbing the aptamer onto the AuNP surfaces to maintain AuNP dispersion under high-salt conditions. Upon target addition, specific aptamer–target binding causes the aptamer to desorb from the AuNPs, leading to salt-induced aggregation and a corresponding color change.

**Figure 6:**
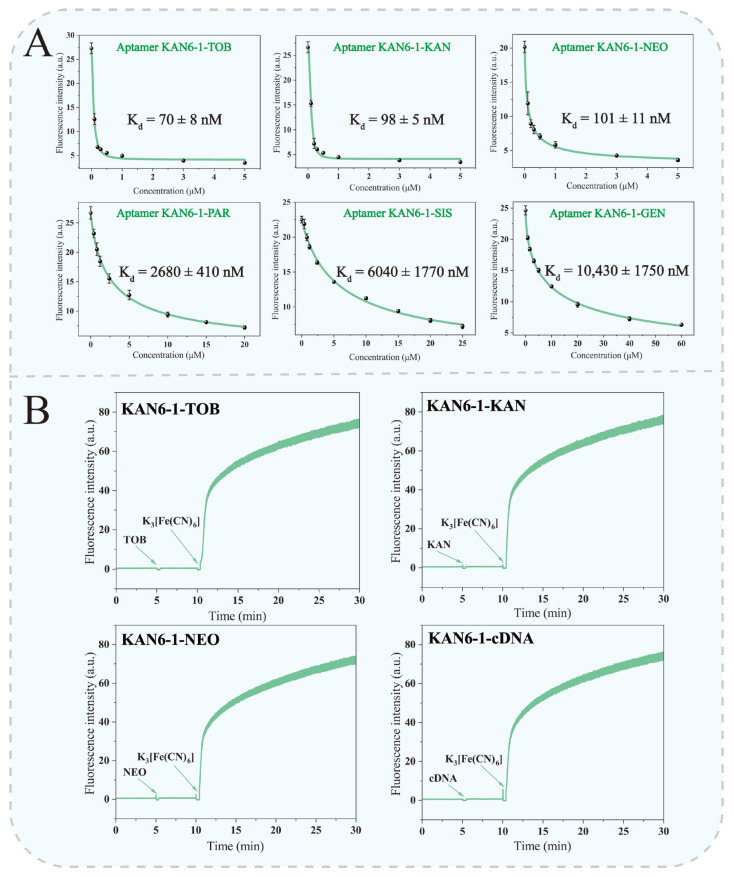
(**A**) To determine the binding affinity of the aptamer for its corresponding targets by ThT fluorescence assay, 20 µL of each target (87.5 µM TOB/NEO/KAN, 350 µM PAR, 437.5 µM SIS, or 1.05 mM GEN) was titrated into a mixture of 313 µL MES buffer, 7 µL of 10 µM aptamer, and 10 µL of 200 µM ThT. K_d_ values were calculated accordingly. (**B**) Kinetic process of the binding of pre-adsorbed KAN6-1 on AuNPs with respective target molecules and cDNA. FAM-labeled KAN6-1 (100 nM) was pre-adsorbed on AuNPs. At 5 min, desorption was attempted by the addition of 40 µL of 100 nM targets or 40 µL of 2.5 µM cDNA. At 10 min, 50 µL of K_3_[Fe(CN)_6_] was added to etch the AuNPs and release the adsorbed FAM-labeled aptamer.

First, we determined the binding ability of aptamer KAN6-1 to its corresponding targets using the ThT fluorescence assay. The K_d_ values for TOB, KAN, and NEO were 70 ± 8, 98 ± 5, and 101 ± 11 nM, respectively (Figure 6A), indicating that this aptamer exhibited good affinity for the aforementioned targets. We therefore selected this aptamer for subsequent studies.

This study employed a FAM-labeled aptamer for testing, enabling direct quantitative evaluation of whether AAs can induce aptamer desorption from the AuNP surfaces by measuring the fluorescence intensity. Initially, AuNPs were pre-incubated with 25, 50, 100, and 200 nM target molecules. The results showed that the adsorption of target molecules on the AuNP surfaces did not hinder subsequent aptamer adsorption (Figure S4). According to the affinity measurements in Figure 6A, three AAs (TOB, KAN, and NEO) with the highest affinities for aptamer KAN6-1 were chosen for the desorption assays. Subsequently, FAM-labeled KAN6-1 was incubated with AuNPs to allow for adsorption, followed by the addition of the target or cDNA for a 5 min reaction, during which changes in the fluorescence intensity of the system were continuously monitored (Figure 6B). The results showed that there was no substantial increase in fluorescence intensity after adding the target, indicating that the target failed to induce desorption of the FAM-labeled aptamer. Subsequently, after adding K_3_[Fe(CN)_6_] to the system, the AuNPs were gradually etched and dissolved, releasing the adsorbed FAM-labeled aptamer, which caused a substantial rise in fluorescence intensity. This result confirmed that the aptamer was indeed pre-adsorbed on the AuNP surfaces, but the target molecules and cDNA could not effectively desorb it. Notably, the phenomenon that aptamers adsorbed on AuNPs are difficult to be desorbed by cDNA has also been reported in the study by Liu et al. [36]. They found that DNA adsorbs onto AuNPs through coordination between the nucleobases and the Au surface, and this interaction is remarkably strong, much stronger than the hybridization between DNA and cDNA. Furthermore, we pre-adsorbed the aptamer onto AuNPs before adding the target molecules (Figure S5). The results revealed that the adsorption of the aptamer on the AuNP surfaces reduced the sensitivity of the AuNPs to AAs, thereby lowering the detection limit.

Furthermore, Liu’s team in 2020 also demonstrated with the KAN aptamer that aptamers adsorbed on AuNPs were barely desorbed by KAN [23]. Their study showed that KAN can adsorb strongly onto the AuNP surfaces and directly induce their aggregation. Likewise, they found that whether aptamers, mutants, or random DNA sequences were used, almost identical color responses were observed in the presence of KAN, indicating that the detection outcome does not depend on the specific recognition of the aptamer. Moreover, fluorescence assay results showed that even high concentrations of KAN could desorb only approximately 12% of the pre-adsorbed aptamers from the AuNP surfaces, further excluding the possibility that KAN releases the aptamers through competitive binding. The above results revealed a substantial limitation of AuNP colorimetric aptasensors in practical applications: even when the aptamer exhibits strong specific affinity for its target, its adsorption on the AuNP surfaces may be extremely stable. Consequently, target binding may fail to trigger effective desorption, making it difficult to achieve the desired detection performance. However, it is noteworthy that numerous studies have successfully detected various targets using this method. In these successful cases, AuNP aggregation is often caused by target molecule-mediated aggregation rather than aptamer desorption from their surfaces.

## 4. Conclusions

In this study, we systematically investigated the interactions of 13 AAs with AuNPs. All tested AAs exhibited strong interactions with AuNPs and considerably affected their colloidal stability. Except for SPE, the other 12 AAs induced AuNP aggregation at relatively low concentrations and markedly reduced their stability in high-salt environments. Furthermore, we observed a red shift in the UV–Vis spectra of the aggregated AuNPs, indicating the occurrence of a plasmon coupling effect.

AuNP aggregation induced by AAs had multiple effects. At high concentrations, AAs directly induced AuNP aggregation; however, at low concentrations that did not cause aggregation, adding low-concentration NaCl also led to AuNP aggregation, indicating that these AAs reduce AuNP salt stability. This occurs because AAs cause partial displacement of surface citrate ions on AuNPs via ligand exchange, which weakens the charge balance. Concurrently, the AAs adsorbed on the AuNP surfaces generate steric hindrance because of their molecular volume, which partially inhibits the complete physical aggregation of AuNPs under high-salt conditions, rendering the aggregation process reversible. Studies based on DLVO theory further confirmed that aggregation induced by AAs occurs at the secondary minimum. Using mPEG–SH (3000 Da), which has an extremely high affinity for Au, to competitively displace AAs created greater steric hindrance, leading to the redispersion of AuNPs. In addition, using DFT calculations to analyze the molecular structure and electronic distribution characteristics of the AAs, we further elucidated their mechanism of action. The lone pair electrons on the nitrogen atoms within the amino groups of AAs can form strong σ-coordinate bonds with the Au surface, thereby promoting their adsorption onto AuNPs. Finally, this study confirmed that target molecules (e.g., TOB, KAN, and NEO) and cDNA cannot effectively induce desorption of the aptamer from the AuNP surfaces, indicating that the interaction between AuNPs and the aptamer is excessively strong. Based on these results, we contend that AuNPs may be unsuitable for constructing simple colorimetric aptasensors for the detection of aminoglycoside antibiotics. The limitations primarily stem from two factors. First, the strong adsorption between AuNPs and the aptamer makes the desorption process difficult to achieve. Second, nonspecific interactions between target molecules and AuNPs can severely interfere with the detection signal.

These findings not only reveal the inherent limitations of AuNPs in constructing colorimetric sensors for AAs, but also, at a deeper level, provide critical warnings and strategic guidance for the in vitro selection (SELEX) process and sensor design of aptamers. First, during the aptamer selection stage, the interaction between the target molecules and AuNPs should be carefully evaluated. Conventional AuNP-based aptamer selection mainly utilizes the competitive binding between targets and AuNPs for nucleic acids, causing specific aptamers to desorb from the AuNP surfaces, and then employs salt-induced aggregation and color change to separate and enrich high-affinity sequences [37]. Our work demonstrates that AAs exhibit strong interactions with AuNPs, which weaken the colloidal stability of AuNPs through ligand exchange. This implies that if such targets are introduced into the selection system, they may preferentially occupy the active sites on AuNP surfaces, thereby not only interfering with the formation of target–aptamer complexes but also generating false-positive signals due to induced AuNP aggregation. Therefore, we strongly recommend that the interfering effects of targets on the dispersion medium be pre-evaluated during the in vitro selection of aptamers to ensure the reliability of experimental results. Second, for the design of colorimetric sensors based on AuNPs, the suitability of the “adsorption–desorption” detection mode should be evaluated in advance. The core principle of these sensors is that target molecules can effectively compete and displace the adsorbed aptamers from the AuNP surfaces, leading to a measurable change in optical signal [38]. However, our experimental results indicate that neither the target molecules nor cDNA can desorb the aptamers from the AuNP surfaces. Therefore, we strongly suggest that researchers must rigorously design control experiments using random sequences or aptamer mutants before adopting such sensing strategies, in order to correct for background signals arising from nonspecific adsorption.

## Data Availability

The original contributions presented in this study are included in the article/Supplementary Materials. Further inquiries can be directed to the corresponding authors.

## Supplementary Materials

The following supporting information can be downloaded at: https://www.mdpi.com/article/10.3390/bios16070388/s1, Figure S1. Transmission Electron Microscopy (TEM) image of the citrate–AuNPs used in this study. Figure S2: (A) Successful preparation of BSPP–AuNPs. The phosphorus atom in BSPP has a much stronger coordination ability with AuNPs than citrate. The successful preparation of BSPP–AuNPs was confirmed by a stability test in the presence of salt: while citrate–AuNPs aggregated, BSPP–AuNPs remained dispersed. (B) Resistance of citrate–AuNPs (left) and BSPP–AuNPs (right) to the 13 AAs. Antibiotic concentrations just sufficient to induce aggregation of citrate-AuNPs were used.; Figure S3: HOMO–LUMO energy levels of the 13 AAs.; Figure S4: Effect of pre-adsorbed AAs on the adsorption of aptamers onto AuNPs. Fluorescence response of FAM-labeled KAN6-1 toward AuNPs pre-adsorbed with varying concentrations of AAs (0–200 nM). After pre-adsorption of AAs, 5 μL of 10 μM FAM-KAN6-1 was added to the mixture for fluorescence measurements.; Figure S5: Inhibition of the interaction between AAs and AuNPs via aptamer modification. 30 µL of 10 µM KAN6-1 was adsorbed onto AuNPs. Then, 50 µL of 200 µM AAs were titrated to a final concentration of 10 µM. For SPE, 50 µL of 20 mM solution was titrated to a final concentration of 1 mM. Table S1: ssDNA sequences used in this study.
