# Supplementary material for: Elucidating the Mechanism of Interactions Between Aminoglycosides and AuNPs: Why the Classical Colorimetric Assay May Falsely Report Aptamer Affinity
Source: Biosensors (Basel). 2026 Jul 17;16(7):388. doi: 10.3390/bios16070388 (PMC13406443; doi:10.3390/bios16070388)
Supplement: Supplementary file 1 [file biosensors-16-00388-s001.zip › biosensors-4436834-supplementary.pdf]

Supplementary Materials

Elucidating the Mechanism of Interactions Between Aminoglycosides and AuNPs: Why the Classical Colorimetric Assay May Falsely Report Aptamer Affinity

Yaning Liang †, Shiyi Fang †, Zhuoer Chen, Yuzhuo Chen, Qingqing Yang, Xuelan Shu and Tao Le *

Chongqing Key Laboratory of Conservation and Utilization of Freshwater Fishes, College of Life Sciences, Chongqing Normal University, Chongqing 401331, China; 2024110513044@stu.cqnu.edu.cn (Y.L.); 2025210513058@stu.cqnu.edu.cn (S.F.); 2025010513002@stu.cqnu.edu.cn (Z.C.); 2024110513031@stu.cqnu.edu.cn (Y.C.); 2024210513076@stu.cqnu.edu.cn (Q.Y.); 2025110513059@stu.cqnu.edu.cn (X.S.)

* Correspondence: letao@cqnu.edu.cn; Tel./Fax: +86-23-65918413

† These authors contributed equally to this work.

Table S1. ssDNA sequences used in this study.

ssDNA	Sequence (5′-3′)
KAN6-1	GACGACGAGGGCCTGAAACTTGCTGGAACGGTTTAAGTCGTC
cDNA (KAN6-1)	GACGACTTAAACCGTTCCAGCAAGTTTCAGGCCCTCGTCGTC

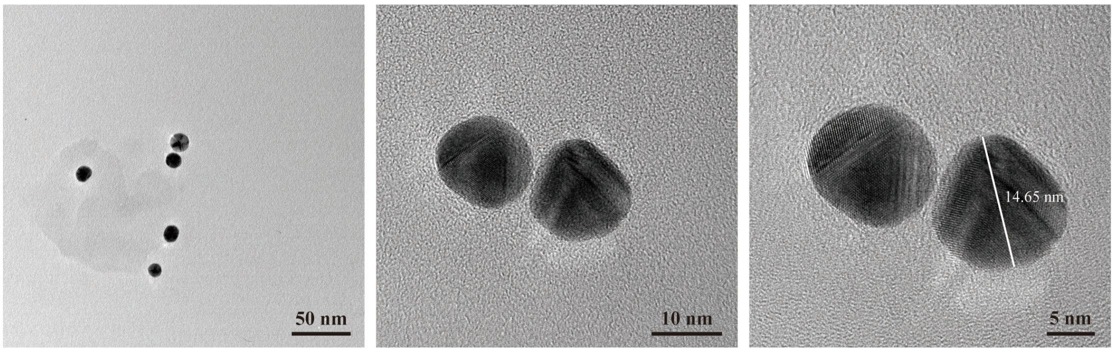

Figure S1. Transmission Electron Microscopy (TEM) image of the citrate–AuNPs used in this study.

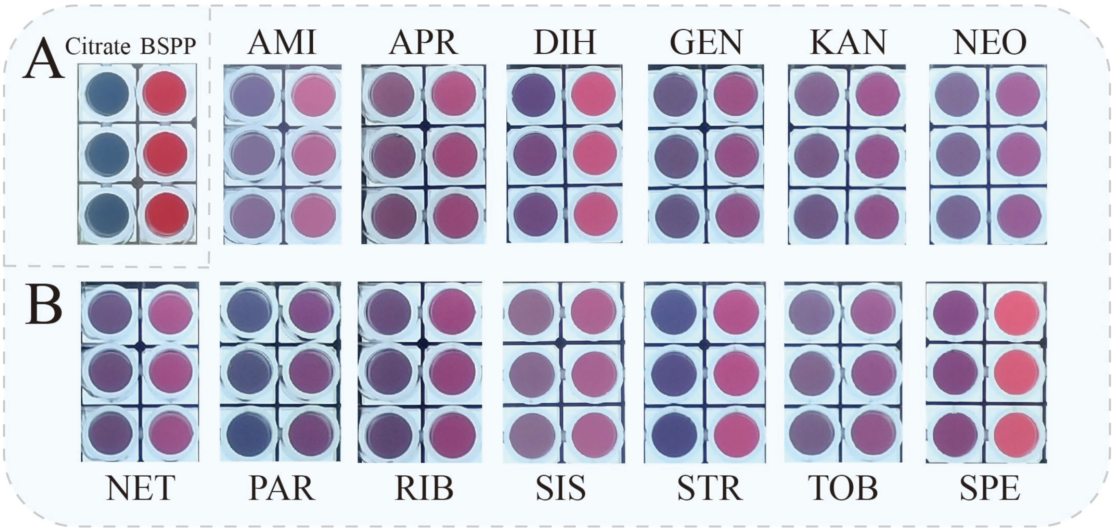

Figure S2. (A) Successful preparation of BSPP–AuNPs. The phosphorus atom in BSPP has a much stronger coordination ability with AuNPs than citrate. The successful preparation of BSPP–AuNPs was confirmed by a stability test in the presence of salt: while citrate–AuNPs aggregated, BSPP–AuNPs remained dispersed. (B) Resistance of citrate–AuNPs (left) and BSPP–AuNPs (right) to the 13 AAs. Antibiotic concentrations just sufficient to induce aggregation of citrate-AuNPs were used.

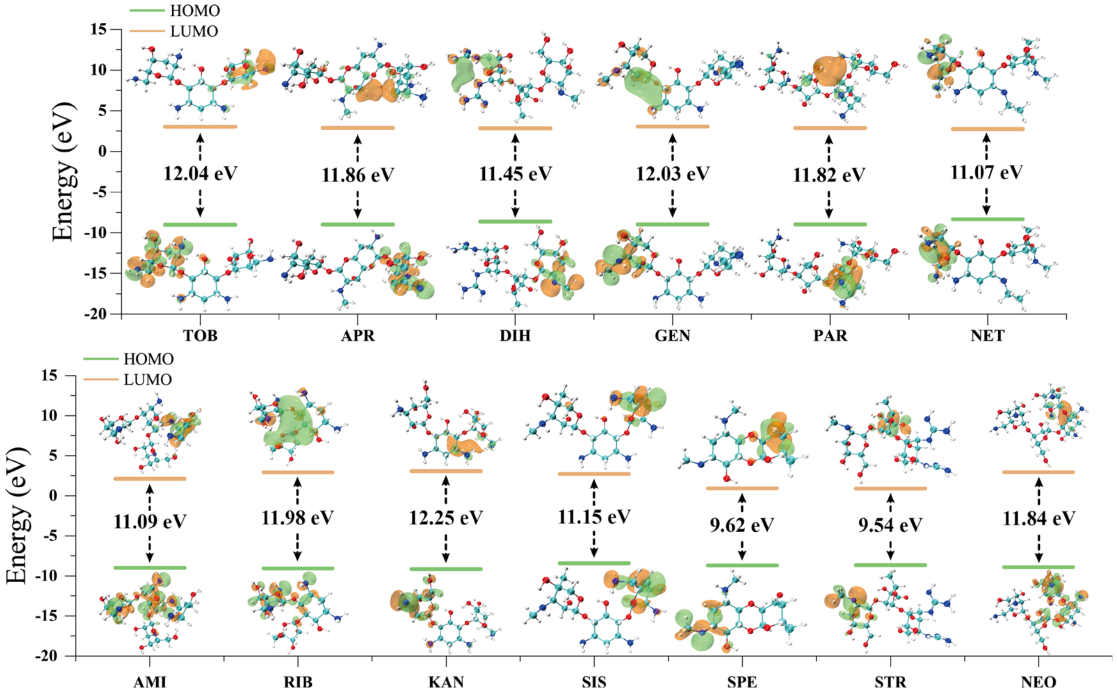

Figure S3. HOMO–LUMO energy levels of the 13 AAs.

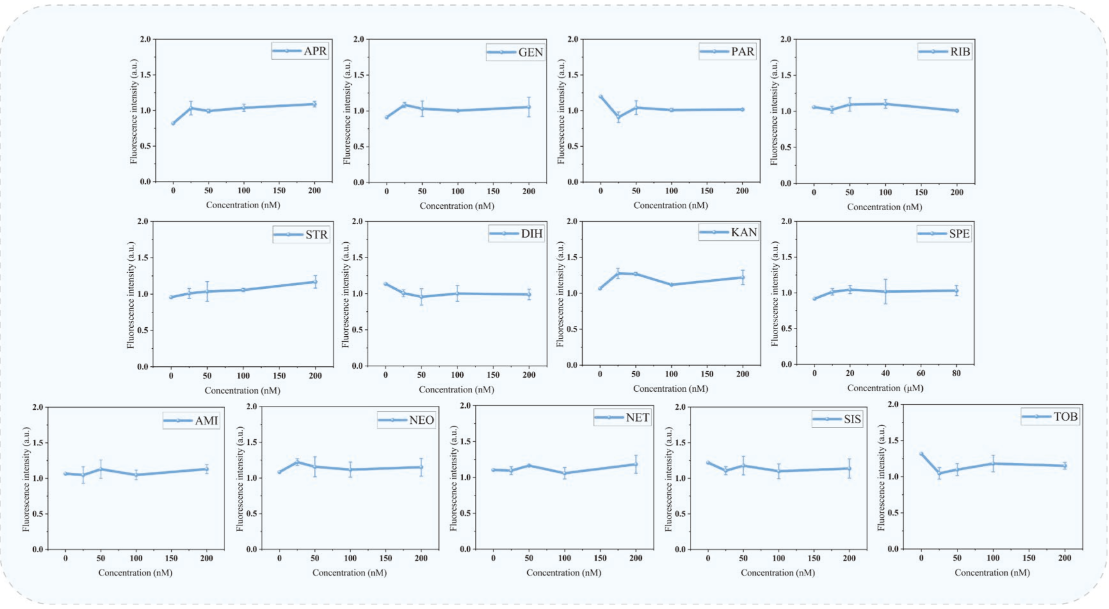

Figure S4. Effect of pre-adsorbed AAs on the adsorption of aptamers onto AuNPs. Fluorescence response of FAM-labeled KAN6-1 toward AuNPs pre-adsorbed with varying concentrations of AAs (0–200 nM). After pre-adsorption of AAs, 5 μL of 10 μM FAM-KAN6-1 was added to the mixture for fluorescence measurements.

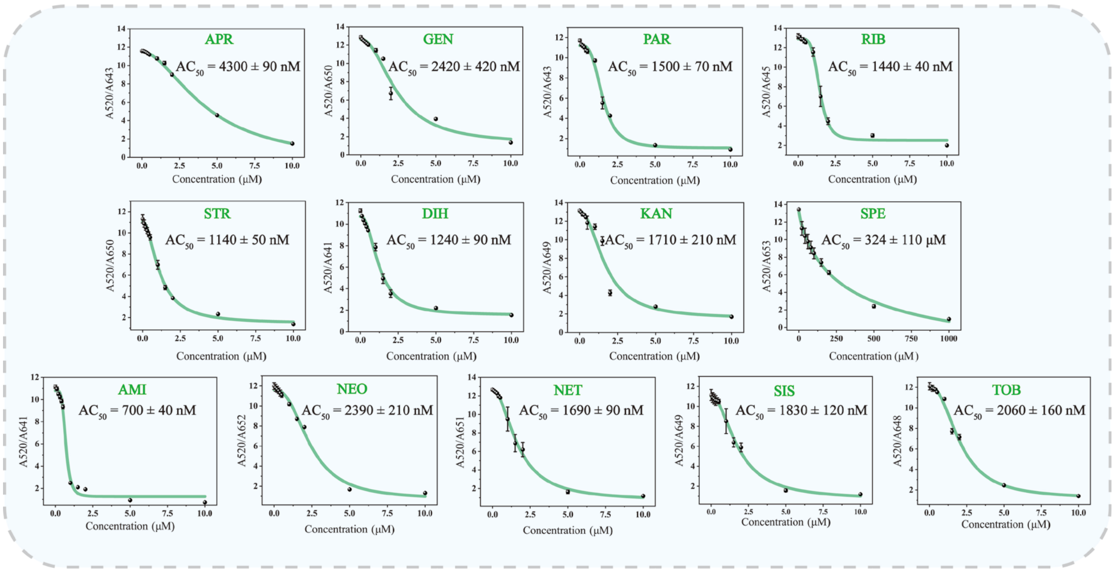

Figure S5. Inhibition of the interaction between AAs and AuNPs via aptamer modification. 30 μL of 10 μM KAN6-1 was adsorbed onto AuNPs. Then, 50 μL of 200 μM AAs were titrated to a final concentration of 10 μM. For SPE, 50 μL of 20 mM solution was titrated to a final concentration of 1 mM.
